# Supplementary figures and images for: Identification of high protein kinase CK2α in HPV(+) oropharyngeal squamous cell carcinoma and correlation with clinical outcomes
Source: PeerJ. 2021 Dec 13;9:e12519. doi: 10.7717/peerj.12519 (PMC8675248; doi:10.7717/peerj.12519)

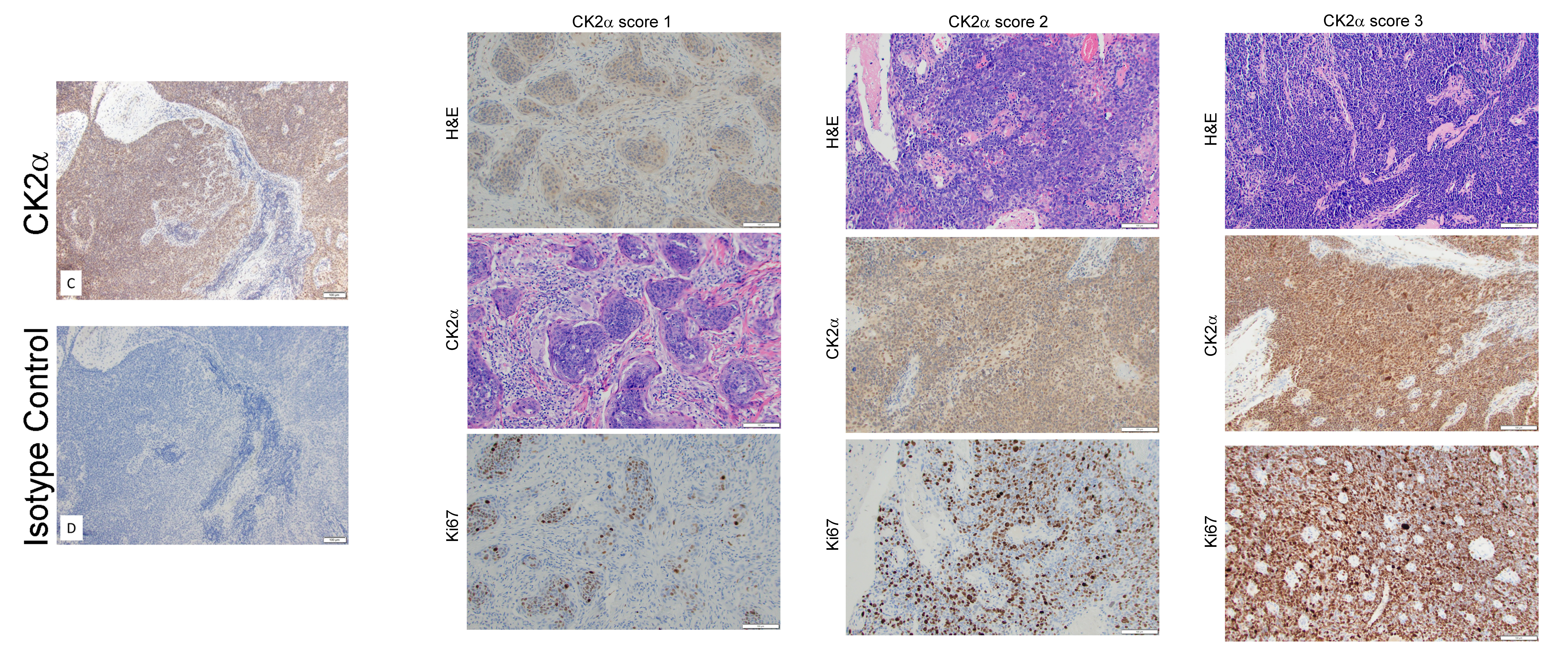

Supplement: Supplemental Information 2 [file peerj-09-12519-s002.jpg]
