# Supplementary material for: Identification of high protein kinase CK2α in HPV(+) oropharyngeal squamous cell carcinoma and correlation with clinical outcomes
Source: PeerJ. 2021 Dec 13;9:e12519. doi: 10.7717/peerj.12519 (PMC8675248; doi:10.7717/peerj.12519)
Supplement: Supplemental Information 3 [file peerj-09-12519-s003.docx]

Analyses summary for association of CK2α scores with variables

| Variable | Total N=119 | one N=28 | two N=60 | three N=31 | P-Value |
| --- | --- | --- | --- | --- | --- |
| **Smoking Status (SMSTATUS)** |  |  |  |  |  |
| Never Smoked | 7 (5.88) | 0 (0) | 4 (6.67) | 3 (9.68) | 0.0646 ^a^ |
| Current Smoker | 58 (48.74) | 19 (67.86) | 29 (48.33) | 10 (32.26) |  |
| Former Smoker | 54 (45.38) | 9 (32.14) | 27 (45) | 18 (58.06) |  |
| N Missing | 0 | 0 | 0 | 0 |  |
| **Gender (GENDER2)** |  |  |  |  |  |
| Male | 119 (100) | 28 (100) | 60 (100) | 31 (100) | 0.0646 ^a^ |
| N Missing | 0 | 0 | 0 | 0 |  |
| **age (AGE)** |  |  |  |  |  |
| Mean | 63.08 | 63.57 | 63.15 | 62.48 | 0.8408 ^b^ |
| Std Dev | 7.16 | 6.87 | 6.87 | 8.12 |  |
| Median | 63.00 | 63.50 | 63.50 | 63.00 |  |
| Minimum | 44.00 | 53.00 | 50.00 | 44.00 |  |
| Maximum | 88.00 | 88.00 | 82.00 | 80.00 |  |
| N Missing | 0 | 0 | 0 | 0 |  |
| **Alcohol Use (ALCOHOL2)** |  |  |  |  |  |
| Non Drinker | 19 (15.97) | 4 (14.29) | 9 (15) | 6 (19.35) | 0.8434 ^a^ |
| Current Drinker | 58 (48.74) | 13 (46.43) | 32 (53.33) | 13 (41.94) |  |
| Former Drinker | 42 (35.29) | 11 (39.29) | 19 (31.67) | 12 (38.71) |  |
| N Missing | 0 | 0 | 0 | 0 |  |
| **Tumor Site; label (TSUBSITE_2)** |  |  |  |  |  |
| Tonsil | 68 (58.62) | 19 (73.08) | 36 (60) | 13 (43.33) | 0.0031 ^a^ |
| Base of Tongue | 41 (35.34) | 3 (11.54) | 21 (35) | 17 (56.67) |  |
| Soft Palate | 6 (5.17) | 3 (11.54) | 3 (5) | 0 (0) |  |
| Pharyngeal Wall | 1 (0.86) | 1 (3.85) | 0 (0) | 0 (0) |  |
| N Missing | 3 | 2 | 0 | 1 |  |
| **T Stage (TSTAGE)** |  |  |  |  |  |
| T1 | 46 (38.66) | 12 (42.86) | 24 (40) | 10 (32.26) | 0.9405 ^a^ |
| T2 | 39 (32.77) | 8 (28.57) | 21 (35) | 10 (32.26) |  |
| T3 | 19 (15.97) | 5 (17.86) | 8 (13.33) | 6 (19.35) |  |
| T4 | 15 (12.61) | 3 (10.71) | 7 (11.67) | 5 (16.13) |  |
| N Missing | 0 | 0 | 0 | 0 |  |
| **N Stage (NSTAGE2)** |  |  |  |  |  |
| N0 | 24 (20.17) | 11 (39.29) | 8 (13.33) | 5 (16.13) | 0.1113 ^a^ |
| N1 | 14 (11.76) | 2 (7.14) | 8 (13.33) | 4 (12.9) |  |
| N2 | 72 (60.5) | 12 (42.86) | 41 (68.33) | 19 (61.29) |  |
| N3 | 9 (7.56) | 3 (10.71) | 3 (5) | 3 (9.68) |  |
| N Missing | 0 | 0 | 0 | 0 |  |
| **Stage Group (AJCCSTAGE_2)** |  |  |  |  |  |
| I | 10 (8.4) | 7 (25) | 3 (5) | 0 (0) | 0.0350 ^a^ |
| II | 9 (7.56) | 2 (7.14) | 5 (8.33) | 2 (6.45) |  |
| III | 16 (13.45) | 3 (10.71) | 8 (13.33) | 5 (16.13) |  |
| IVA | 73 (61.34) | 12 (42.86) | 41 (68.33) | 20 (64.52) |  |
| IVB | 11 (9.24) | 4 (14.29) | 3 (5) | 4 (12.9) |  |
| N Missing | 0 | 0 | 0 | 0 |  |
| **Inital Treatment Only (TX_MODALITY_INIT)** |  |  |  |  |  |
| CCRT concurrent chemo radiation | 69 (57.98) | 13 (46.43) | 40 (66.67) | 16 (51.61) | 0.5361 ^a^ |
| XRT radiation alone | 12 (10.08) | 5 (17.86) | 4 (6.67) | 3 (9.68) |  |
| SUR/CCRT surgery and chemo_rad | 9 (7.56) | 2 (7.14) | 4 (6.67) | 3 (9.68) |  |
| SUR surgery alone | 12 (10.08) | 5 (17.86) | 5 (8.33) | 2 (6.45) |  |
| SUR/XRT surgery and adjuvant radiation | 6 (5.04) | 1 (3.57) | 3 (5) | 2 (6.45) |  |
| CCRT/SURG chemo_rad and sugery | 11 (9.24) | 2 (7.14) | 4 (6.67) | 5 (16.13) |  |
| N Missing | 0 | 0 | 0 | 0 |  |
| **P16 (P16)** |  |  |  |  |  |
| negative | 35 (29.66) | 21 (75) | 12 (20) | 2 (6.67) | <0.0001 ^c^ |
| positive | 83 (70.34) | 7 (25) | 48 (80) | 28 (93.33) |  |
| N Missing | 1 | 0 | 0 | 1 |  |
| **Two Category HPV E6E7 (DICHHPVE6E7)** |  |  |  |  |  |
| negative | 35 (29.41) | 22 (78.57) | 10 (16.67) | 3 (9.68) | <0.0001 ^c^ |
| positive | 84 (70.59) | 6 (21.43) | 50 (83.33) | 28 (90.32) |  |
| N Missing | 0 | 0 | 0 | 0 |  |
| **KI 67 Index (KI67)** |  |  |  |  |  |
| <10 low | 6 (5.04) | 5 (17.86) | 1 (1.67) | 0 (0) | 0.0003 ^c^ |
| 10-20 borderline | 27 (22.69) | 11 (39.29) | 11 (18.33) | 5 (16.13) |  |
| >20 high | 86 (72.27) | 12 (42.86) | 48 (80) | 26 (83.87) |  |
| N Missing | 0 | 0 | 0 | 0 |  |
| **Did disease progress? (DISESEPROG2)** |  |  |  |  |  |
| no | 78 (65.55) | 15 (53.57) | 41 (68.33) | 22 (70.97) | 0.3030 ^c^ |
| yes | 41 (34.45) | 13 (46.43) | 19 (31.67) | 9 (29.03) |  |
| N Missing | 0 | 0 | 0 | 0 |  |
| **Progression Free Survival Days (PFSURVDAYS)** |  |  |  |  |  |
| Mean | 1175.78 | 998.86 | 1108.43 | 1465.94 | 0.2062 ^b^ |
| Std Dev | 1088.99 | 1029.89 | 976.71 | 1307.12 |  |
| Median | 834.00 | 666.50 | 834.50 | 1025.00 |  |
| Minimum | 0.00 | 57.00 | 0.00 | 83.00 |  |
| Maximum | 4201.00 | 3795.00 | 4076.00 | 4201.00 |  |
| N Missing | 0 | 0 | 0 | 0 |  |
| **Death (DEATH)** |  |  |  |  |  |
| no | 77 (64.71) | 11 (39.29) | 42 (70) | 24 (77.42) | 0.0044 ^c^ |
| yes | 42 (35.29) | 17 (60.71) | 18 (30) | 7 (22.58) |  |
| N Missing | 0 | 0 | 0 | 0 |  |
| **Overall Survival Days (OVSURVTIMEDAYS)** |  |  |  |  |  |
| Mean | 1330.49 | 1137.46 | 1253.15 | 1654.52 | 0.1260 ^b^ |
| Std Dev | 1060.50 | 1026.71 | 944.69 | 1250.83 |  |
| Median | 918.00 | 794.00 | 962.00 | 1072.00 |  |
| Minimum | 71.00 | 71.00 | 91.00 | 252.00 |  |
| Maximum | 4201.00 | 3795.00 | 4076.00 | 4201.00 |  |
| N Missing | 0 | 0 | 0 | 0 |  |

^a^ Fisher's Exact test

^b^ ANOVA Type III F-test

^c^ Pearson's Chi-Square

Relationship between p16 and hpve6e7

| \| **Table of p16 by HPV_E6E7** \| \| \| \| \| \| \| --- \| --- \| --- \| --- \| --- \| --- \| \| **p16(p16)** \| **HPV_E6E7(HPV_E6E7)** \| \| \| \| \| \| **neg** \| **pos1+** \| **pos2+** \| **pos3+** \| **Total** \| \| **negative** \| \| 33 \| \| --- \| \| 27.97 \| \| 94.29 \| \| 94.29 \| \| \| 2 \| \| --- \| \| 1.69 \| \| 5.71 \| \| 10.53 \| \| \| 0 \| \| --- \| \| 0.00 \| \| 0.00 \| \| 0.00 \| \| \| 0 \| \| --- \| \| 0.00 \| \| 0.00 \| \| 0.00 \| \| \| 35 \| \| --- \| \| 29.66 \| \|  \| \|  \| \| \| **positive** \| \| 2 \| \| --- \| \| 1.69 \| \| 2.41 \| \| 5.71 \| \| \| 17 \| \| --- \| \| 14.41 \| \| 20.48 \| \| 89.47 \| \| \| 39 \| \| --- \| \| 33.05 \| \| 46.99 \| \| 100.00 \| \| \| 25 \| \| --- \| \| 21.19 \| \| 30.12 \| \| 100.00 \| \| \| 83 \| \| --- \| \| 70.34 \| \|  \| \|  \| \| \| **Total** \| \| 35 \| \| --- \| \| 29.66 \| \| \| 19 \| \| --- \| \| 16.10 \| \| \| 39 \| \| --- \| \| 33.05 \| \| \| 25 \| \| --- \| \| 21.19 \| \| \| 118 \| \| --- \| \| 100.00 \| \| \| **Frequency Missing = 1** \| \| \| \| \| \| |
| --- | --- | --- | --- | --- | --- | --- | --- | --- | --- | --- | --- | --- | --- | --- | --- | --- | --- | --- | --- | --- | --- | --- | --- | --- | --- | --- | --- | --- | --- | --- | --- | --- | --- | --- | --- | --- | --- | --- | --- | --- | --- | --- | --- | --- | --- | --- | --- | --- | --- | --- | --- | --- | --- | --- | --- | --- | --- | --- | --- | --- | --- | --- | --- | --- | --- | --- | --- | --- | --- | --- | --- | --- | --- | --- | --- | --- | --- | --- | --- | --- | --- | --- | --- | --- | --- | --- | --- | --- | --- | --- | --- |

| **Statistics for Table of p16 by HPV_E6E7** |
| --- |

| **Statistic** | **DF** | **Value** | **Prob** |
| --- | --- | --- | --- |
| **Chi-Square** | 3 | 100.3844 | <.0001 |
| **Likelihood Ratio Chi-Square** | 3 | 115.3606 | <.0001 |
| **Mantel-Haenszel Chi-Square** | 1 | 76.4872 | <.0001 |
| **Phi Coefficient** |  | 0.9223 |  |
| **Contingency Coefficient** |  | 0.6780 |  |
| **Cramer's V** |  | 0.9223 |  |

Univariate and Multivariate Cox Regression

Highlighted in pink – p value

Highlighted in blue –HR and CI

**HPV**

| **Type 3 Tests** | | | |
| --- | --- | --- | --- |
| **Effect** | **DF** | **Wald Chi-Square** | **Pr > ChiSq** |
| **dichhpve6e7** | 1 | 13.2258 | 0.0003 |

| **Analysis of Maximum Likelihood Estimates** | | | | | | | | |
| --- | --- | --- | --- | --- | --- | --- | --- | --- |
| **Parameter** |  | **DF** | **Parameter Estimate** | **Standard Error** | **Chi-Square** | **Pr > ChiSq** | **Hazard Ratio** | **Label** |
| **dichhpve6e7** | **negative** | 1 | 1.14031 | 0.31355 | 13.2258 | 0.0003 | 3.128 | Two Category HPV E6E7 negative |

| **Hazard Ratios for Two Category HPV E6E7** | | | |
| --- | --- | --- | --- |
| **Description** | **Point Estimate** | **95% Wald Confidence Limits** | |
| **dichhpve6e7 negative vs positive** | 3.128 | 1.692 | 5.783 |

**HPV controlling for smoking (HPV remains significant)**

| **Type 3 Tests** | | | |
| --- | --- | --- | --- |
| **Effect** | **DF** | **Wald Chi-Square** | **Pr > ChiSq** |
| **dichhpve6e7** | 1 | 7.8803 | 0.0050 |
| **newdichsm** | 1 | 2.2623 | 0.1326 |

| **Analysis of Maximum Likelihood Estimates** | | | | | | | | |
| --- | --- | --- | --- | --- | --- | --- | --- | --- |
| **Parameter** |  | **DF** | **Parameter Estimate** | **Standard Error** | **Chi-Square** | **Pr > ChiSq** | **Hazard Ratio** | **Label** |
| **dichhpve6e7** | **negative** | 1 | 0.94284 | 0.33587 | 7.8803 | 0.0050 | 2.567 | Two Category HPV E6E7 negative |
| **newdichsm** |  | 1 | 0.53582 | 0.35624 | 2.2623 | 0.1326 | 1.709 |  |

| **Hazard Ratios for Two Category HPV E6E7** | | | |
| --- | --- | --- | --- |
| **Description** | **Point Estimate** | **95% Wald Confidence Limits** | |
| **dichhpve6e7 negative vs positive** | 2.567 | 1.329 | 4.959 |

**CK2**

| **Type 3 Tests** | | | |
| --- | --- | --- | --- |
| **Effect** | **DF** | **Wald Chi-Square** | **Pr > ChiSq** |
| **dichck2ihc** | 1 | 8.5504 | 0.0035 |

| **Analysis of Maximum Likelihood Estimates** | | | | | | | | |
| --- | --- | --- | --- | --- | --- | --- | --- | --- |
| **Parameter** |  | **DF** | **Parameter Estimate** | **Standard Error** | **Chi-Square** | **Pr > ChiSq** | **Hazard Ratio** | **Label** |
| **dichck2ihc** | **2 and 3 high** | 1 | -0.92181 | 0.31524 | 8.5504 | 0.0035 | 0.398 | Two category CK2 IHC 2 and 3 high |

| **Hazard Ratios for Two category CK2 IHC** | | | |
| --- | --- | --- | --- |
| **Description** | **Point Estimate** | **95% Wald Confidence Limits** | |
| **dichck2ihc 2 and 3 high vs low** | 0.398 | 0.214 | 0.738 |

**CK2 controlling for smoking (CK2 remains significant)**

| **Type 3 Tests** | | | |
| --- | --- | --- | --- |
| **Effect** | **DF** | **Wald Chi-Square** | **Pr > ChiSq** |
| **dichck2ihc** | 1 | 5.7776 | 0.0162 |
| **newdichsm** | 1 | 4.5151 | 0.0336 |

| **Analysis of Maximum Likelihood Estimates** | | | | | | | | |
| --- | --- | --- | --- | --- | --- | --- | --- | --- |
| **Parameter** |  | **DF** | **Parameter Estimate** | **Standard Error** | **Chi-Square** | **Pr > ChiSq** | **Hazard Ratio** | **Label** |
| **dichck2ihc** | **2 and 3 high** | 1 | -0.77354 | 0.32182 | 5.7776 | 0.0162 | 0.461 | Two category CK2 IHC 2 and 3 high |
| **newdichsm** |  | 1 | 0.71932 | 0.33853 | 4.5151 | 0.0336 | 2.053 |  |

| **Hazard Ratios for Two category CK2 IHC** | | | |
| --- | --- | --- | --- |
| **Description** | **Point Estimate** | **95% Wald Confidence Limits** | |
| **dichck2ihc 2 and 3 high vs low** | 0.461 | 0.246 | 0.867 |

**Ki67**

| **Type 3 Tests** | | | |
| --- | --- | --- | --- |
| **Effect** | **DF** | **Wald Chi-Square** | **Pr > ChiSq** |
| **dichki67** | 1 | 1.9778 | 0.1596 |

| **Analysis of Maximum Likelihood Estimates** | | | | | | | | |
| --- | --- | --- | --- | --- | --- | --- | --- | --- |
| **Parameter** |  | **DF** | **Parameter Estimate** | **Standard Error** | **Chi-Square** | **Pr > ChiSq** | **Hazard Ratio** | **Label** |
| **dichki67** | **<=20 low or borderline** | 1 | 0.46175 | 0.32834 | 1.9778 | 0.1596 | 1.587 | dichki67 <=20 low or borderline |

| **Hazard Ratios for dichki67** | | | |
| --- | --- | --- | --- |
| **Description** | **Point Estimate** | **95% Wald Confidence Limits** | |
| **dichki67 <=20 low or borderline vs >20 high** | 1.587 | 0.834 | 3.020 |

**Age (continuous so for 1 yr increase)**

| **Analysis of Maximum Likelihood Estimates** | | | | | | | |
| --- | --- | --- | --- | --- | --- | --- | --- |
| **Parameter** | **DF** | **Parameter Estimate** | **Standard Error** | **Chi-Square** | **Pr > ChiSq** | **Hazard Ratio** | **Label** |
| **age** | 1 | 0.01059 | 0.02073 | 0.2609 | 0.6095 | 1.011 | age |

| **Hazard Ratios for age** | | | |
| --- | --- | --- | --- |
| **Description** | **Point Estimate** | **95% Wald Confidence Limits** | |
| **age Unit=1** | 1.011 | 0.970 | 1.053 |

**Dichotomous alcohol (current or former drinker vs non-drinker)**

| **Type 3 Tests** | | | |
| --- | --- | --- | --- |
| **Effect** | **DF** | **Wald Chi-Square** | **Pr > ChiSq** |
| **dichalc** | 1 | 0.9180 | 0.3380 |

| **Analysis of Maximum Likelihood Estimates** | | | | | | | | |
| --- | --- | --- | --- | --- | --- | --- | --- | --- |
| **Parameter** |  | **DF** | **Parameter Estimate** | **Standard Error** | **Chi-Square** | **Pr > ChiSq** | **Hazard Ratio** | **Label** |
| **dichalc** | **current or former drinker** | 1 | 0.50400 | 0.52603 | 0.9180 | 0.3380 | 1.655 | dichalc current or former drinker |

| **Hazard Ratios for dichalc** | | | |
| --- | --- | --- | --- |
| **Description** | **Point Estimate** | **95% Wald Confidence Limits** | |
| **dichalc current or former drinker vs non drinker** | 1.655 | 0.590 | 4.641 |

**tstage**

| **Type 3 Tests** | | | |
| --- | --- | --- | --- |
| **Effect** | **DF** | **Wald Chi-Square** | **Pr > ChiSq** |
| **dichtstage** | 1 | 0.6525 | 0.4192 |

| **Analysis of Maximum Likelihood Estimates** | | | | | | | | |
| --- | --- | --- | --- | --- | --- | --- | --- | --- |
| **Parameter** |  | **DF** | **Parameter Estimate** | **Standard Error** | **Chi-Square** | **Pr > ChiSq** | **Hazard Ratio** | **Label** |
| **dichtstage** | **tstage 3 or 4** | 1 | 0.26522 | 0.32833 | 0.6525 | 0.4192 | 1.304 | dichtstage tstage 3 or 4 |

| **Hazard Ratios for dichtstage** | | | |
| --- | --- | --- | --- |
| **Description** | **Point Estimate** | **95% Wald Confidence Limits** | |
| **dichtstage tstage 3 or 4 vs tstage 1 or 2** | 1.304 | 0.685 | 2.481 |

**Nstage**

| **Type 3 Tests** | | | |
| --- | --- | --- | --- |
| **Effect** | **DF** | **Wald Chi-Square** | **Pr > ChiSq** |
| **nstadich** | 1 | 0.3395 | 0.5601 |

| **Analysis of Maximum Likelihood Estimates** | | | | | | | | |
| --- | --- | --- | --- | --- | --- | --- | --- | --- |
| **Parameter** |  | **DF** | **Parameter Estimate** | **Standard Error** | **Chi-Square** | **Pr > ChiSq** | **Hazard Ratio** | **Label** |
| **nstadich** | **1 2 or 3** | 1 | 0.23012 | 0.39494 | 0.3395 | 0.5601 | 1.259 | nstadich 1 2 or 3 |

| **Hazard Ratios for nstadich** | | | |
| --- | --- | --- | --- |
| **Description** | **Point Estimate** | **95% Wald Confidence Limits** | |
| **nstadich 1 2 or 3 vs none** | 1.259 | 0.580 | 2.730 |

**AJCC (per 1 unit increase – continuous)**

| **Analysis of Maximum Likelihood Estimates** | | | | | | | |
| --- | --- | --- | --- | --- | --- | --- | --- |
| **Parameter** | **DF** | **Parameter Estimate** | **Standard Error** | **Chi-Square** | **Pr > ChiSq** | **Hazard Ratio** | **Label** |
| **ajccstage_2** | 1 | 0.14141 | 0.15392 | 0.8441 | 0.3582 | 1.152 | Stage Group |

| **Hazard Ratios for Stage Group** | | | |
| --- | --- | --- | --- |
| **Description** | **Point Estimate** | **95% Wald Confidence Limits** | |
| **ajccstage_2 Unit=1** | 1.152 | 0.852 | 1.558 |

**Txmod_init (Treatment)**

| **Type 3 Tests** | | | |
| --- | --- | --- | --- |
| **Effect** | **DF** | **Wald Chi-Square** | **Pr > ChiSq** |
| **txmod_init** | 4 | 4.0964 | 0.3931 |

| **Analysis of Maximum Likelihood Estimates** | | | | | | | | |
| --- | --- | --- | --- | --- | --- | --- | --- | --- |
| **Parameter** |  | **DF** | **Parameter Estimate** | **Standard Error** | **Chi-Square** | **Pr > ChiSq** | **Hazard Ratio** | **Label** |
| **txmod_init** | **CCRT concurrent chemo radiation** | 1 | 0.26248 | 0.53999 | 0.2363 | 0.6269 | 1.300 | txmod_init CCRT concurrent chemo radiation |
| **txmod_init** | **SUR/CCRT surgery and chemo_rad** | 1 | -0.53793 | 0.65516 | 0.6741 | 0.4116 | 0.584 | txmod_init SUR/CCRT surgery and chemo_rad |
| **txmod_init** | **SUR/XRT surgery and adjuvant radiation** | 1 | -0.82558 | 1.12212 | 0.5413 | 0.4619 | 0.438 | txmod_init SUR/XRT surgery and adjuvant radiation |
| **txmod_init** | **XRT radiation alone** | 1 | 0.34156 | 0.67373 | 0.2570 | 0.6122 | 1.407 | txmod_init XRT radiation alone |

| **Hazard Ratios for txmod_init** | | | |
| --- | --- | --- | --- |
| **Description** | **Point Estimate** | **95% Wald Confidence Limits** | |
| **txmod_init CCRT concurrent chemo radiation vs SUR surgery alone** | 1.300 | 0.451 | 3.747 |
| **txmod_init SUR/CCRT surgery and chemo_rad vs SUR surgery alone** | 0.584 | 0.162 | 2.109 |
| **txmod_init SUR/XRT surgery and adjuvant radiation vs SUR surgery alone** | 0.438 | 0.049 | 3.950 |
| **txmod_init XRT radiation alone vs SUR surgery alone** | 1.407 | 0.376 | 5.270 |

**Smoking - current vs. former or never**

| **Type 3 Tests** | | | |
| --- | --- | --- | --- |
| **Effect** | **DF** | **Wald Chi-Square** | **Pr > ChiSq** |
| **newdichsm** | 1 | 6.7075 | 0.0096 |

| **Analysis of Maximum Likelihood Estimates** | | | | | | | | |
| --- | --- | --- | --- | --- | --- | --- | --- | --- |
| **Parameter** |  | **DF** | **Parameter Estimate** | **Standard Error** | **Chi-Square** | **Pr > ChiSq** | **Hazard Ratio** | **Label** |
| **newdichsm** | **current smoker** | 1 | 0.85836 | 0.33143 | 6.7075 | 0.0096 | 2.359 | newdichsm current smoker |

| **Hazard Ratios for newdichsm** | | | |
| --- | --- | --- | --- |
| **Description** | **Point Estimate** | **95% Wald Confidence Limits** | |
| **newdichsm current smoker vs never or former smoker** | 2.359 | 1.232 | 4.517 |
